# Coronary microvascular disease in hypertrophic and infiltrative cardiomyopathies

**DOI:** 10.1007/s12350-022-03040-2

**Published:** 2022-08-01

**Authors:** Andreas A. Giannopoulos, Ronny R. Buechel, Philipp A. Kaufmann

**Affiliations:** Department of Nuclear Medicine, Cardiac Imaging, University Hospital and University Zurich, Raemistrasse 100, 8091 Zurich, Switzerland

**Keywords:** Coronary microvascular disease, CMD, left ventricular hypertrophy, coronary flow reserve, cardiac PET, CMR

## Abstract

Pathologic hypertrophy of the cardiac muscle is a commonly encountered phenotype in clinical practice, associated with a variety of structural and non-structural diseases. Coronary microvascular disease is considered to play an important role in the natural history of this pathological phenotype. Non-invasive imaging modalities, most prominently positron emission tomography and cardiac magnetic resonance, have provided insights into the pathophysiological mechanisms of the interplay between hypertrophy and the coronary microvasculature. This article summarizes the current knowledge on coronary microvascular dysfunction in the most frequently encountered forms of pathologic hypertrophy.

**Supplementary Information:**

The online version contains supplementary material available at 10.1007/s12350-022-03040-2.

## Introduction

Cardiac hypertrophy refers to an increase in the mass of the left or the right ventricle or both as an adaptive mechanism to pressure and/or volume stress, genetic mutations of several proteins, and/or loss of contractile mass as a result of prior infarction.^1^ Most commonly encountered in clinical practice is left ventricular hypertrophy (LVH), defined as an increase of the left ventricular myocardial mass resulting from increased wall thickness or an increase in the cavitary dimensions, or both. LVH can be primary or secondary and characterizes a wide variety of cardiac structural and non-structural disease-entities such as ischemic heart disease, arterial hypertension, heart failure, valvular disease, and a range of genetical cardiomyopathies. The estimation of LV wall thickness and mass in routine clinical practice is derived from measurements obtained by echocardiography or cardiac magnetic resonance (CMR).

Coronary microvascular disease (CMD), although not the hallmark and the primary mechanism for the pathogenesis of pathological hypertrophy, is considered to hold a critical role in the natural history of this pathological phenotype. Several pathophysiological mechanisms have been proposed and although our understanding remains incomplete, in most cases, CMD in LVH is multifactorial (Table 1). Patients with pathological hypertrophy in the absence of relevant epicardial coronary artery disease (CAD) are categorized as CMD type B (myocardial disease without CAD), characterized by capillary rarefaction and adverse remodeling of intramural coronary arterioles due to medial wall thickening (leading to an increased wall-to-lumen ratio).^2^ This remodeling process commonly affects the entirety of the left ventricle, indicating a more diffuse nature of the disease.^3^ CMD in LVH is considered to confer important clinical and prognostic implications. Non-invasive imaging modalities allowing for assessment of myocardial blood flow (MBF) and myocardial flow reserve (MFR; defined as hyperemic(stress)/rest MBF) have provided valuable insights on CMD in pathological LVH. Myocardial perfusion positron emission tomography (PET) which is considered the gold standard for routine clinical MBF assessment, but also CMR and contrast-enhanced transthoracic echocardiography (contrast-TTE) have been shown to be valuable tools in both clinical and research settings.

**Table 1:** Potential mechanisms of CMD in pathologic LVH phenotypes

Hypetrophic phenotype	Potential CMD mechanisms
HCM	Reduced capillary density Extravascular compression Vascular remodeling Smooth muscle cells dysfunction Endothelial dysfunction Fibrosis
Anderson–Fabry’s disease	Vascular wall infiltration Vascular remodeling Endothelial dysfunction Proliferation of smooth muscle cells Fibrosis
Sarcoidosis	Inflammation Endothelial dysfunction Fibrosis
Cardiac amyloidosis	Vascular wall infiltration and thickening Extravascular compression Luminal obstruction Endothelial dysfunction

This review aims to summarize the current knowledge on CMD in the most commonly encountered LVH-phenotypes in clinical practice.

## Specific clinical hypertrophy phenotypes

### Hypertrophic cardiomyopathy

Hypertrophic cardiomyopathy (HCM) is the most common monogenic cardiovascular disease, inherited through an autosomal dominant pattern.^4^ HCM is commonly morphologically characterized by asymmetrical hypertrophy of the left ventricle, with a predilection for the interventricular septum and histologically by hypertrophy of cardiac myocytes, myocyte disarray, interstitial fibrosis, and thickening and narrowing of the intramural coronary arteries.^5,6^ Given the several secondary etiologies of LVH, diagnosis of HCM should be established in the absence of other cardiac, systemic, or metabolic factors or diseases resulting in a hypertrophic phenocopy while at the same time, genetical testing if available is essential in the diagnostic process.^4,7^ Impaired MBF during stress and its sequelae, myocardial ischemia is considered an essential feature of HCM patients, and altered microvascular function is associated with poor outcomes.^8,9^ Compared to patients without pathological hypertrophy, HCM patients have significantly reduced hyperemic MBF and MFR while resting MBF is preserved.^10–12^ Clinically, a significant association has been reported between pathological MFR and symptoms (primarily angina), risk of sustained ventricular arrhythmia, progression to end-stage heart failure, and cardiovascular death.^8,13–16^

**Figure 1:**
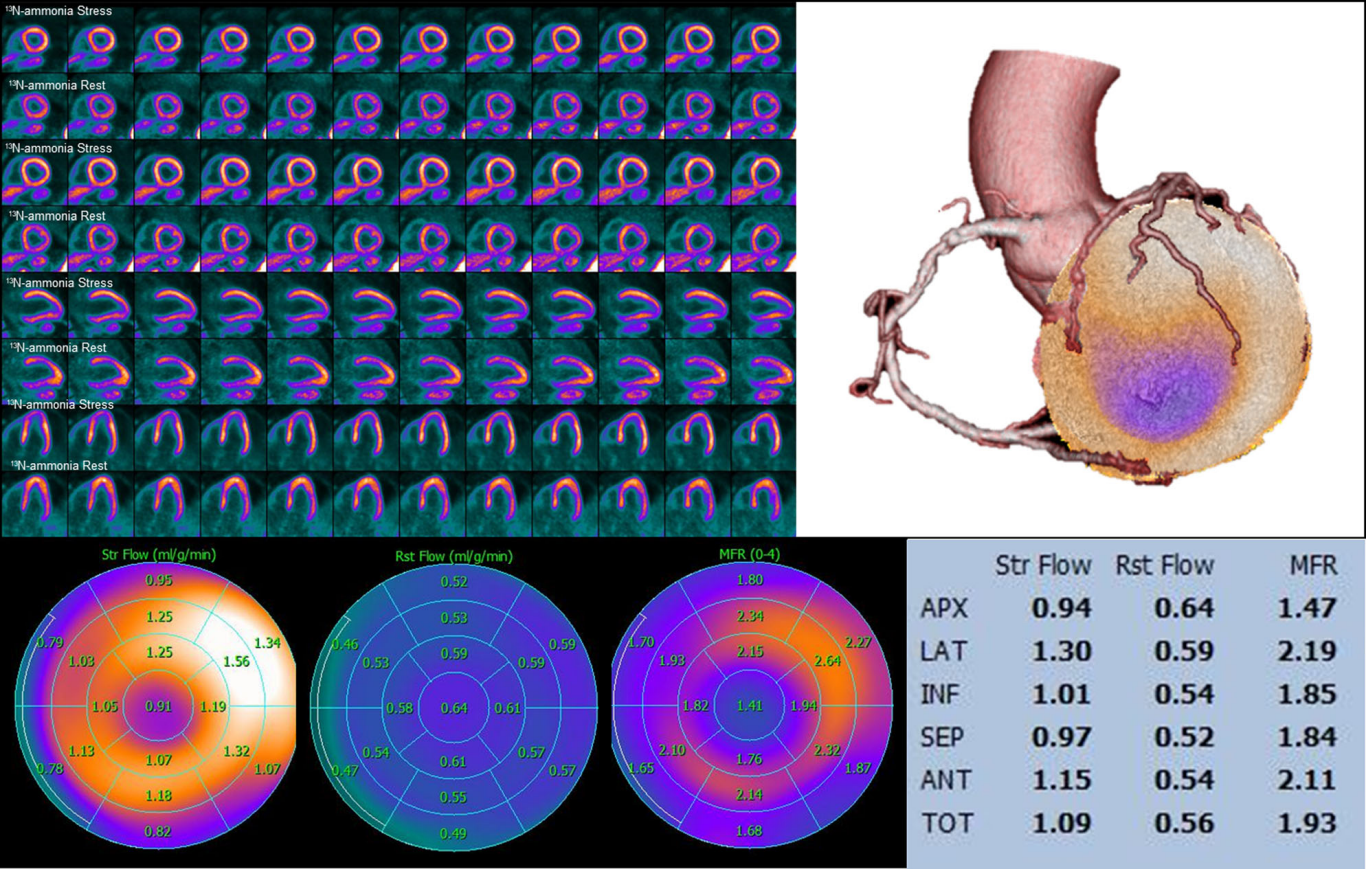
CMD in a 66 years old patient with apical HCM. Left upper Panel shows ^13^N-ammonia PET perfusion study (short axis, horizontal and vertical long axis slices) with evidence of subendocardial perfusion defect in stress which did not correspond to a coronary artery supply territory as assessed in fused coronary computed tomography angiography and ^13^N-ammonia PET images (right upper panel). Lower panel shows quantitative PET analysis with evidence of CMD depicted by globally pathological MFR (Total MFR 1.93). The apical segments show markedly reduced hyperemic MBF and MFR while the microcirculature is also compromised on the non-hypertrophic inferior and septal LV-segments

The etiology of CMD in HCM is multifactorial, including reduced capillary density and vascular remodeling, fibrosis, myocyte disarray, and extravascular compression.^10,17,18^ The latter contributes to endothelial dysfunction, which in turn leads to inadequate hyperemic MBF response predisposing for myocardial ischemia.^10^ Remodeling of intramural coronary arterioles increases coronary vascular resistance, and it seems to affect the entirety of the myocardium, even remote regions that are not hypertrophied.^9,17,19^ Increased loading conditions and wall stress in these left ventricles with severely impaired diastolic dysfunction as well as the increased metabolic demand of the hypertrophied myocardium further contribute to the presence and extent of CMD, particularly in the subendocardium.^9^ Several studies have shown that MBF is more blunted in the subendocardium and in the more hypertrophied areas, but without always sparing of the non-hypertrophied segments.^16,19^ (Figure 1)

The prevalence of CAD is no different in patients with versus without HCM.^20^ Accounting for the younger age that HCM is diagnosed at and particularly prior to manifestation of hemodynamically significant CAD, CMD is the key determinant of myocardial perfusion abnormalities. Chronic and recurrent myocardial ischemia may lead to replacement fibrosis, which will eventually further precipitate CMD. This pathophysiologic concept might be applicable in most, but not all HCM patients since fibrosis (i.e., late gadolinium enhancement (LGE), at CMR) has been shown to be present without CMD in some patients.^21^ In the majority of patients, myocardial segments without fibrosis have normal global MFR without regional heterogeneity of flow, whereas in the presence of LGE, the majority of symptomatic HCM patients exhibit impaired global MFR with significantly lower hyperemic MBF within fibrotic versus non-fibrotic segments.^21,22^

Similar to the clinical nature of the disease and the phenotypical heterogeneity (localization, extent of hypertrophy, and presence or absence of fibrosis) a substantial heterogeneity in MFR reduction and CMD is observed in HCM patients, potentially owning also to the time-point of the diagnosis and the time of assessment of the microvasculature. While some patients present with normal MFR, others exhibit severely reduced MFR (consistent with significant CMD).^12,23^ Genetical factors might also play a role, in view of the results from a ^13^N-ammonia PET and CMR study showing significantly lower peak MBF in patients with sarcomere myofilament mutations compared to matched genotype-negative patients.^24^

HCM patients with obstructive physiology and elevated left ventricular outflow tract (LVOT) gradients appear to have a worse prognosis compared to those with non-obstructive disease.^25^ Studies assessing the coronary microsiculation in patients with LVOT obstruction have shown contradictory results. A ^13^N-ammonia PET study comparing 11 non-obstructive patients, 12 obstructive patients, and 10 with latent-obstruction (defined as a provoked-LVOT gradient of > 30 mmHg), found no significant correlation between LVOT gradients and global parameters of ischemia or hyperemic MBF and MFR on PET, when comparing symptomatic patients with obstructive versus non-obstructive HCM.^23^ Other groups using ^15^O-water PET showed an inverse correlation between LVOT gradients and hyperemic MBF.^8,10,26^ These discrepant findings could potentially be explained by the labile nature of LVOT obstruction in HCM; a highly variable echocardiographic finding heavily dependent on a myriad of factors, including volume status, autonomic nervous system, physical position, and others.^7^

It has further been shown that myocardial wall thickness is the strongest predictor for reduced global hyperemic MBF and MFR, while MBF decreased in proportion to the increase in end-diastolic wall thickness.^10,23^ Petersen et al. demonstrated that hyperemic MBF decreased by .011 mL⋅min^−1^⋅g^−1^ for each millimeter increase of end-diastolic wall thickness.^27^ Similarly, pathology studies in HCM patients have demonstrated that the percentage of luminal narrowing of intramural coronary small arteries inversely correlated with heart weight and myocyte size and also that there was an inverse relationship between normalized coronary arteriolar lumen and degree of LV hypertrophy.^18,28^ CMR studies in HCM patients, utilizing cardiac diffusion tensor imaging for in vivo characterization of myocardial microstructure, demonstrated that even in myocardial segments with normal wall thickness, normal perfusion, and without scar, diffusion is more isotropic than in controls, suggesting the presence of underlying cardiomyocyte disarray.^29^ In the same population, hyperemic MBF and MFR were reduced, particularly within the subendocardium, not only in hypertrophied and scarred segments but also in segments with normal wall thickness. Transmural perfusion gradient (TPG) is a relative novel quantitative PET metric defined as the ratio of endocardial-to-epicardial MBF. In hypertrophic left ventricles, TPG is decreased during vasodilator-induced hyperemia, and in HCM patients, a reduction of TPG under stress indicates that the impairment of MBF affects primarily the endocardium.^30,31^ In HCM, impaired TPG is related to pathological LVEF stress response, potentially suggesting that the stress-induced subendocardial ischemia may also cause transient LV dysfunction.^32^

Being the most frequently encountered form of pathological LVH, with an estimated prevalence of 1 out of every 200 adults (0.5%), HCM represents a very heterogeneous population whereby CMD is present in several stages of the disease with important clinical and prognostic implications.^33^ The benefit of implementing an assessment of the microvasculature in the individual management of HCM patients remains to be elucidated by future studies.

## Anderson–Fabry’s disease

**Figure 2:**
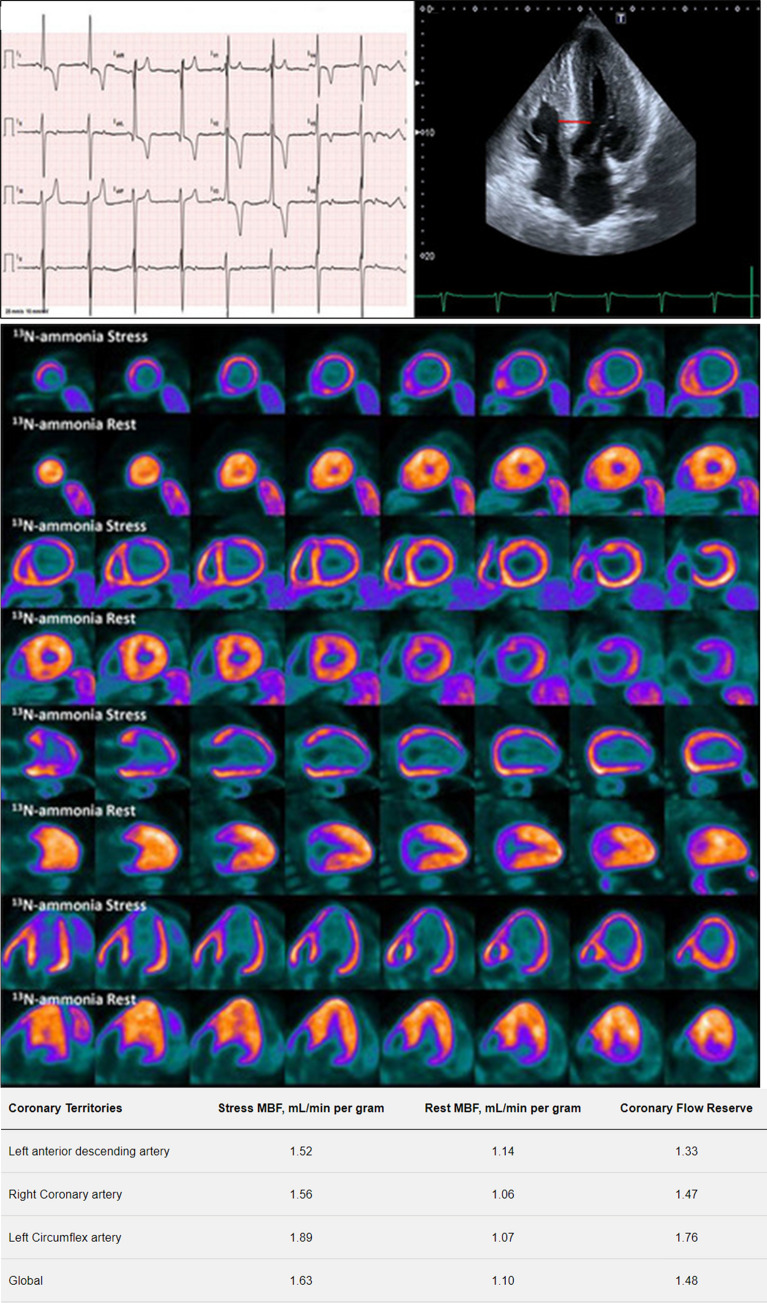
Severe CMD in AFD cardiomyopathy. Upper panel shows electrocardiogram (ECG) (left) and two-dimensional TTE (right), ECG: the 12-lead ECG shows sinus bradycardia, short PR interval and left ventricular hypertrophy (LVH) with deep T wave inversion in the anterolateral leads. Echocardiogram: from the apical 4 chamber view severe concentric LVH is evident with maximal wall thickness of 26 mm at the septum (red line). Right ventricular hypertrophy is also present. Mid panel shows ^13^N-ammonia PET images, displayed in short axis, and vertical axis, and horizontal long axis. No significant perfusion defects are noted at rest. However, during pharmacological stress, severe global subendocardial ischemia is seen. Lower panel shows the quantitative PET analysis with severely reduced global and regional MFR (normal values >2.5). Reproduced with permission from Ref. 44

An X-linked lysosomal storage syndrome, Anderson–Fabry’s disease (AFD) is caused by mutations in the gene encoding α-galactosidase A, resulting in inability to break down glycosphingolipids, which in turn leads to accumulation in multiple organs with subsequent renal, cardiac, and cerebrovascular injury.^34^ The estimated prevalence of AFD in patients with unexplained LVH in adults is around .5% to 1%.^35–37^ Cardiac deposition of globotriaosylceramide in myocytes, vascular endothelium, and smooth muscle cells leads to myocardial ischemia, myocardial wall thickening, and progressively to interstitial replacement fibrosis.^38–40^ These alterations increase coronary vascular resistance and myocardial oxygen demand, which along with direct endothelial cell dysfunction due to globotriaosylceramide storage, nitric oxide pathway dysregulation, and microvascular remodeling are considered to mediate CMD in patients with AFD.^41,42^ In fact, CMD is considered to play an important role in AFD patients, in some representing the first manifestation of cardiac involvement, in others it is responsible for anginal symptoms.^42–44^ (Figure 2)

CMD in AFD has been investigated primarily in PET studies.^42,43,45^ Compared to healthy controls, AFD patients seem to have reduced MFR and hyperemic MBF, irrespective of LVH or gender.^42^ Although AFD is an X-linked disease with males primarily being affected, most heterozygous females also express clinical manifestations, including cardiac involvement, but usually at a later age.^46,47^ CMD might be the earliest manifestation of cardiac involvement, seen even prior to any signs of LVH.^42^ Similarly, reduced hyperemic MBF is inversely related to the degree of LV wall thickness and to age in males, potentially corroborating the hypothesis that CMD precedes the development of LVH. Multiparametric CMR imaging has been used to describe the several stages of AFD, including the initial globotriaosylceramide storage phase characterized by low native T1 mapping values, and followed by the LVH stage with focal and global inflammation leading to fibrosis.^48–51^ At CMR, a reduction in hyperemic MBF has also been shown to be present early in the disease course and was demonstrated to be related with the severity of LVH, as well with regional inflammation and fibrosis.^52^ A CMR study in 114 AFD patients revealed that compared to matched, healthy controls, the patients had significantly lower MBF even prior to the occurrence of LVH and reduction of native T1 mapping values.^53^ Supporting the findings from PET studies, CMD was shown to be present not only prior to hypertrophic phenotype but is also preceding the storage phase of cardiac involvement.

Assessment of the microvasculature function could have implications for the management of AFD patients: Early recognition of cardiac involvement could potentially prompt early initiation of disease-specific treatment that includes either intravenous enzyme replacement therapy (ERT; recombinant α-galactosidase A) or oral chaperon therapy (Migalastat), or even cardiac supportive therapies.^54^ This concept was assessed in PET-based studies with discrepant results, potentially owing to sample sizes too small to assess any ERT response effect.^43,55^ Technological advancements and increased availability of imaging modalities could allow for better evaluation of the effect of therapeutic options on the microvascular function and the potential long-term benefit.

## Sarcoidosis

Sarcoidosis is a systemic inflammatory disease characterized by the formation of non-caseating granulomas upon an unknown trigger in genetically predisposed individuals.^56^ Primarily affecting the lungs and intrathoracic lymph nodes, cardiac involvement is present in a considerable amount of sarcoidosis patients, ranging from 5% to 10% of individuals with systemic manifestations, albeit the exact prevalence is most probably underestimated.^57,58^ Myocardial involvement in cardiac sarcoidosis (CS), phenotypically described as a chameleon-disease, is usually focal or scattered and commonly affects the free lateral LV wall or the basal intraventricular septum, that appears thinned, while in some cases it can result in LVH mimicking HCM.^59–61^ Atrioventricular conductance disturbances, ventricular arrhythmias, and progressive heart failure are the most common clinical manifestations and CS patients may present with angina symptoms even in the absence of significant epicardial CAD. The latter has been attributed to CMD, although this hypothesis up till recently was based on qualitative assessment of myocardial perfusion (with single-photon emission computed tomography) and not on quantification of MBF.^62–65^

A retrospective, combined ^13^N-ammonia and ^18^F-fluorodeoxyglucose (FDG) PET study has provided insights on the relationship between cardiac inflammation and MBF in CS patients.^66^ Sarcoid-mediated inflammation was shown to be associated with regional impairment of MFR. Although resting MBF were found to be within normal limits, irrespective of the presence of inflammation or not, hyperemic MBF and MFR were significantly lower in inflamed myocardium compared to non-inflamed regions. In a small portion of the study participants, assessment of treatment-response on the microcirculation was also evaluated, whereby those that did not respond to immunosuppressive treatment (defined as unchanged or increased myocardial inflammation) had a further decline in hyperemic MBF and MFR. In contrast, response to steroid therapy was associated with preserved coronary circulatory function.^66,67^

Active inflammation in CS patients has also been associated with enhanced coronary vasoconstrictive reactivity at the epicardial and microvascular level, while medical treatment (combined anti-inflammatory and calcium channel blockers) was shown to reduce epicardial spasm effectively.^68^ Work utilizing contrast-TTE reported that in the absence of traditional risk factors for CAD, sarcoidosis patients without known cardiac involvement had a lower MFR as compared to healthy controls.^69^ The mechanism of CMD in CS patients is undoubtedly multifactorial. An early systematic inflammatory state affects the microvascular endothelium. It is considered to result in decreased nitric oxide production, which in turn impairs vasodilation response, particularly in actively inflamed myocardial regions.^70,71^ In advanced stages, a more global and diffuse impairment of MBF might also be present and potentially precedes the phase of structural myocardial changes, including fibrosis and scarring that leads to overt heart failure.^66^ Whether CMD in CS is a by-stander and to what extent it confers prognostic or therapeutic implications remains to be elucidated.

## Cardiac amyloidosis

Amyloidosis is a collection of systemic disorders characterized by the extracellular deposition of insoluble fibrils composed of misfolded proteins in multiple organs.^72^ Cardiac amyloidosis (CA) is caused by cardiac amyloid fibril deposition and the main forms clinically encountered (termed after the precursor protein of the amyloid deposit) include light-chain amyloidosis (AL) or transthyretin-types amyloidosis (ATTR; wild-type-ATTR and mutant-ATTR).^73,74^ Amyloid can be deposited in almost all cardiac structures from pericardium, epi-/endocardium to cardiac valves, myocardium and cardiac vasculature. Both left and right ventricular myocardium can be infiltrated, resulting in biventricular hypertrophy (usually symmetrical in AL and asymmetrical in ATTR) that progresses to a restrictive form of cardiomyopathy.^74^ Deposition of amyloid leads to structural alterations predominantly within the subendocardial and midwall regions ultimately resulting in replacement fibrosis.^75^ Pathological hypertrophy of the ventricles may impede subendocardial perfusion due to capillary rarefaction and compression.^76^ Although significant luminal obstruction of the epicardial coronary arteries rarely occurs, direct vascular deposition of fibrils can further precipitate microvascular obstruction. Functional abnormalities, including autonomic dysfunction and endothelial dysfunction, that characterize amyloidosis are also considered to play a significant role in CMD in CA patients.

**Figure 3:**
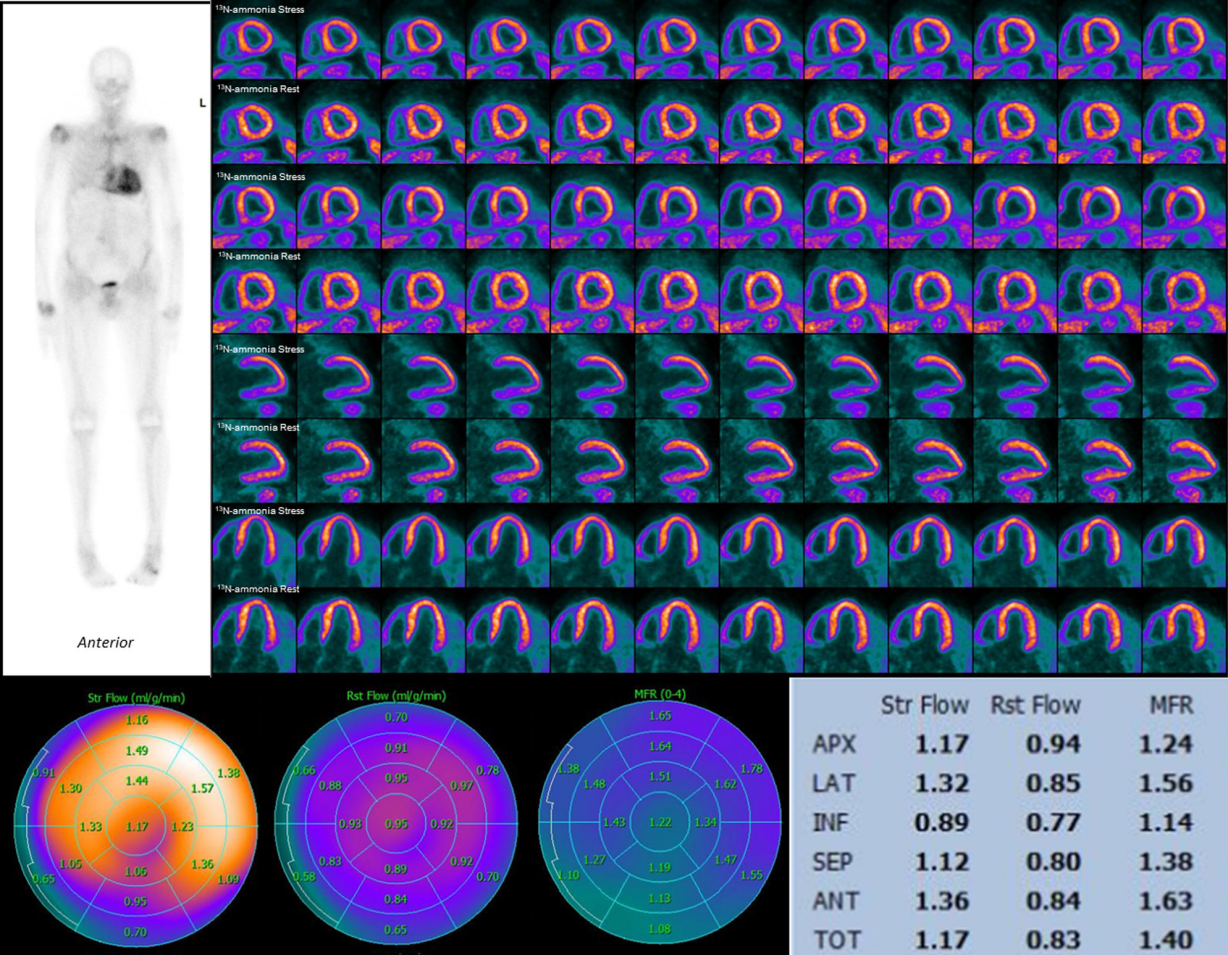
ATTR Amyloidosis and CMD. A 82-year-old patient with left ventricular hypertrophy on TTE was assessed for presence of cardiac amyloidosis. Upper left panel depicts planar whole-body scintigraphy with a bone tracer (Tc-99m-DPD) revealing increased myocardial tracer uptake consistent with cardiac ATTR amyloidosis. Upper right panel shows ^13^N-ammonia PET perfusion images (short axis, horizontal and vertical long axis slices) demonstrating no regional perfusion defect in stress or rest. Quantitative PET analysis shows a significant reduction of hyperemic MBF and a severely reduced global MFR (lower panels)

The last decade has heralded a paradigm shift in the field of CA, given the advent of disease-modifying therapeutic options (primarily for ATTR) such as stabilizing molecules (tafamidis) and genetic silencers (patisiran and inotersen) which have been shown to improve quality of life.^77–80^ Although most studies have focused on the non-bioptic identification of cardiac amyloid depositions, a few smaller studies have also assessed the microvasculature in CA using all available imaging modalities.^76,81–85^ In patients with both AL-CA and ATTR-CA and in the absence of epicardial CAD that were assessed with ^13^N-ammonia PET, rest MBF, hyperemic MBF and MFR were significantly lower when compared to a matched hypertensive patients’ group with LVH.^76^ (Figure 3). Coronary vascular resistance defined as the ratio of mean arterial pressure to MBF at rest (maximal coronary vascular resistance) and peak hyperemia (minimal coronary vascular resistance) was higher in the CA group and was also shown to be increased irrespective of the amyloid subtype. The magnitude of CMD in the CA patients was more severe than in previously reported data in dilated cardiomyopathy and in patients with AFD cardiomyopathy.^42,86^ T1 mapping and extracellular volume as derived from CMR allow for myocardial tissue characterization and have been implemented in a recent work that compared CA patients with HCM patients and healthy controls while also evaluating CMD.^82^ The latter was appraised with the myocardial transit-time (MyoTT), defined as the blood circulation time from the orifice of the coronary arteries to the pooling in the coronary sinus. This surrogate marker of CMD was significantly prolonged in CA patients compared with the HCM and the healthy controls.^82,87^ Real-time contrast TTE with flash bubble destruction has also been used for quantification of MBF in AL CA patients, whereby significant reduction of hyperemic MBF was observed.^85^ Even in patients with rare renal amyloid A (AA) amyloidosis, MFR was significantly lower at contrast-TTE compared to a group of individuals with non-amyloid chronic inflammatory disease and healthy controls.^81^ As cardiac involvement in AA amyloidosis usually occurs at later stages of the disease, assessment of microvascular function could potentially enable earlier identification of patients at risk.

## Conclusion

Current knowledge indicates a close association and interplay between the coronary microvasculature and pathologic myocardial hypertrophy while the prevalence of patients with a hypertrophic phenotype is increasing in clinical practice. Multimodality imaging approaches for non-invasive quantification of MBF provide a distinctive opportunity to evaluate the function of the coronary microcirculation. Technical refinements and wider availability of said diagnostic tools hold the promise to allow for a better understanding of the pathophysiological mechanism and the related clinical and prognostic implications. Finally, integration of patient- and disease status-specific functional assessment of the microvasculature in the diagnostic approach may pave the way for better risk-stratification and patient-tailored therapeutic options.

## Supplementary Information

Below is the link to the electronic supplementary material.

Supplementary file1 (PPTX 905 kb)

## Abbreviations

AFD: Anderson–Fabry’s disease
CA: Cardiac amyloidosis
CMD: Coronary microvascular disease
CMR: Cardiac magnetic resonance
CS: Cardiac sarcoidosis
HCM: Hypertrophic cardiomyopathy
LVH: Left ventricular hypertrophy
MBF: Myocardial blood flow
MFR: Myocardial flow reserve
PET: Positron emission tomography
