# Supplementary material for: Coronary microvascular disease in hypertrophic and infiltrative cardiomyopathies
Source: J Nucl Cardiol. 2022 Aug 1;30(2):800–10. doi: 10.1007/s12350-022-03040-2 (PMC10125945; doi:10.1007/s12350-022-03040-2)
Supplement: Supplementary file 1 — Supplementary file1 (PPTX 905 kb) [file 12350_2022_3040_MOESM1_ESM.pptx]

## Slide 1
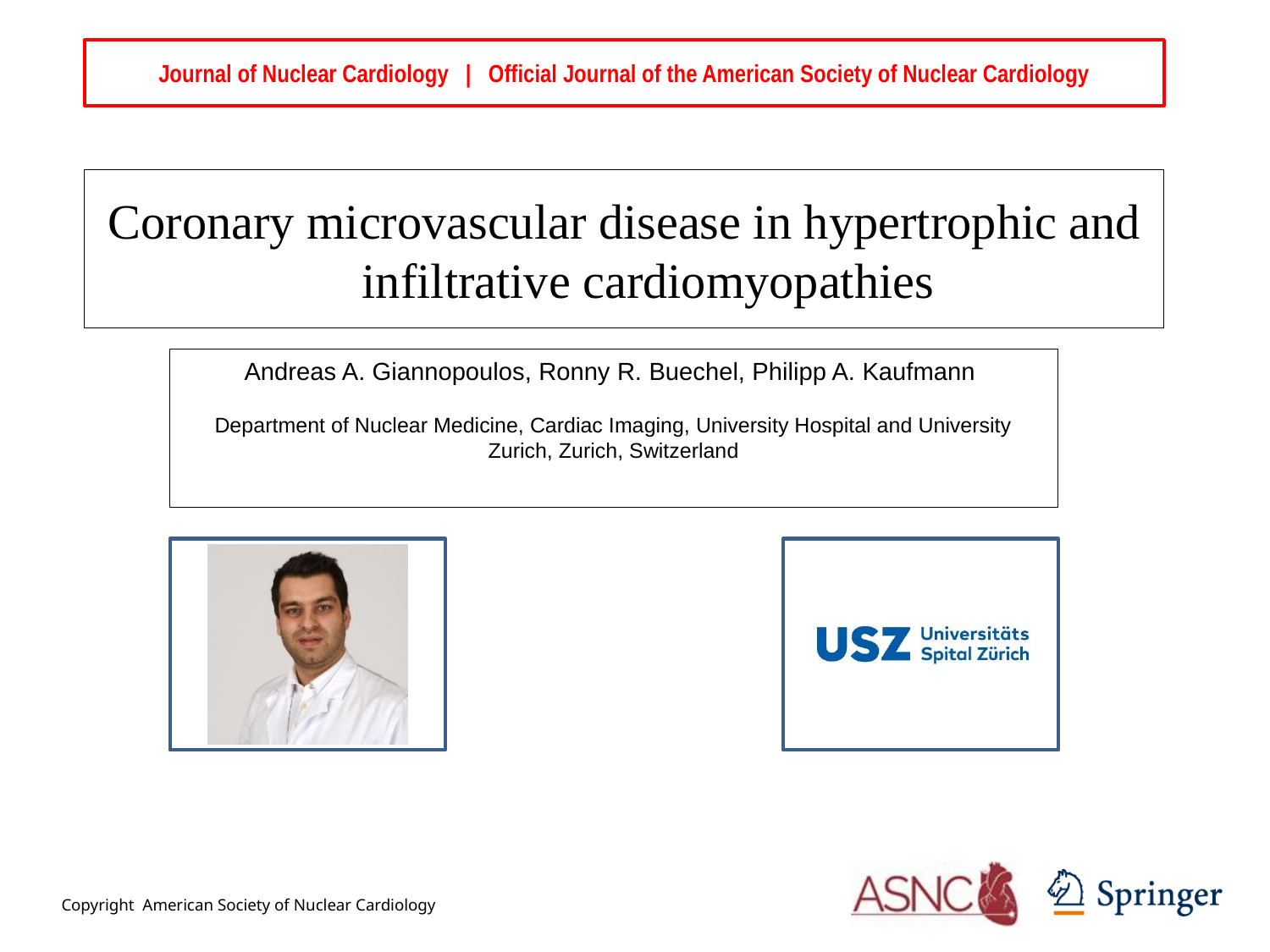

Journal of Nuclear Cardiology | Official Journal of the American Society of Nuclear Cardiology
# Coronary microvascular disease in hypertrophic and infiltrative cardiomyopathies
Andreas A. Giannopoulos, Ronny R. Buechel, Philipp A. Kaufmann
Department of Nuclear Medicine, Cardiac Imaging, University Hospital and University Zurich, Zurich, Switzerland
Head shot of author
required
Copyright American Society of Nuclear Cardiology

## Slide 2
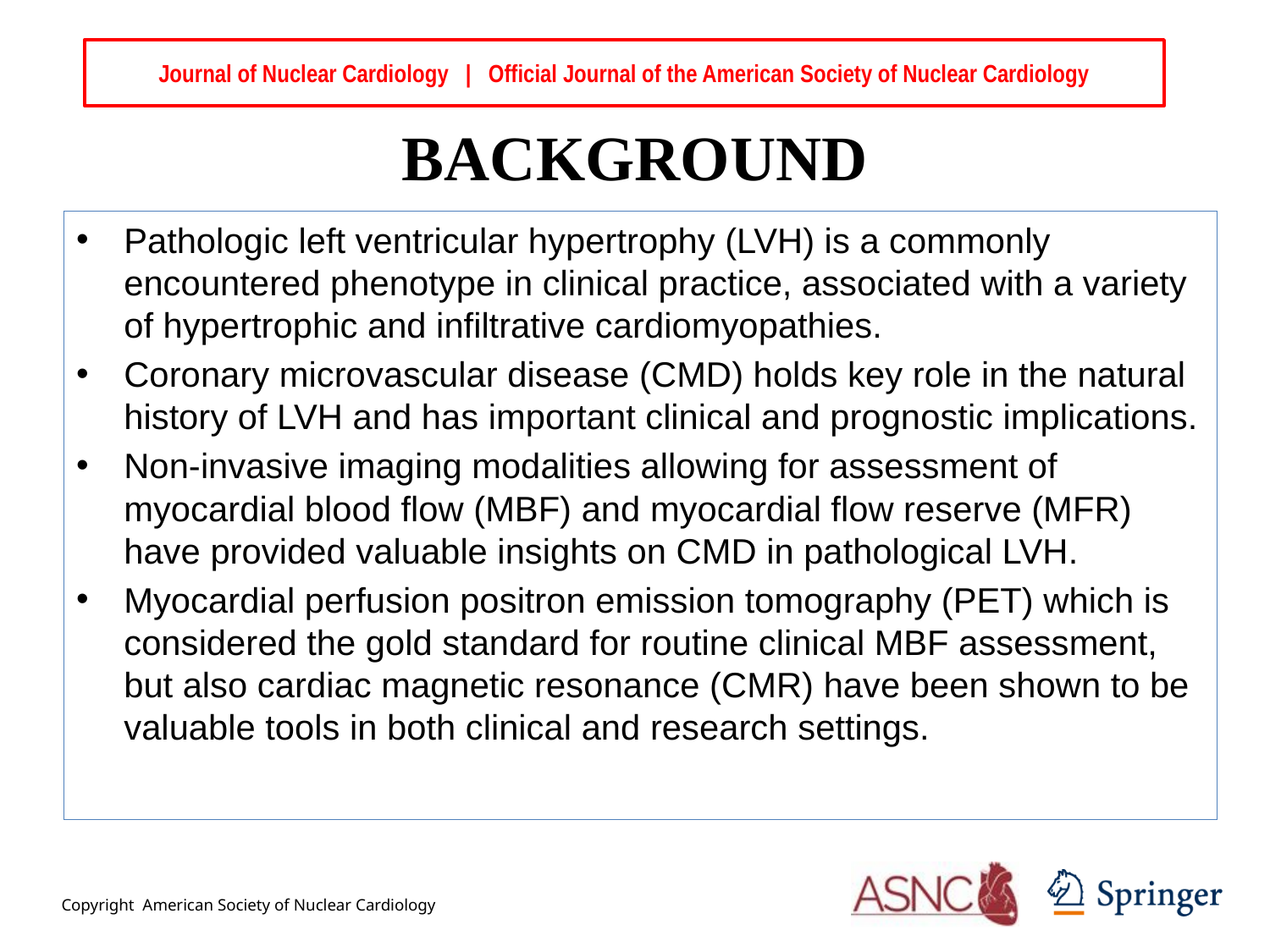

Journal of Nuclear Cardiology | Official Journal of the American Society of Nuclear Cardiology
# BACKGROUND
Pathologic left ventricular hypertrophy (LVH) is a commonly encountered phenotype in clinical practice, associated with a variety of hypertrophic and infiltrative cardiomyopathies.
Coronary microvascular disease (CMD) holds key role in the natural history of LVH and has important clinical and prognostic implications.
Non-invasive imaging modalities allowing for assessment of myocardial blood flow (MBF) and myocardial flow reserve (MFR) have provided valuable insights on CMD in pathological LVH.
Myocardial perfusion positron emission tomography (PET) which is considered the gold standard for routine clinical MBF assessment, but also cardiac magnetic resonance (CMR) have been shown to be valuable tools in both clinical and research settings.
Copyright American Society of Nuclear Cardiology

## Slide 3
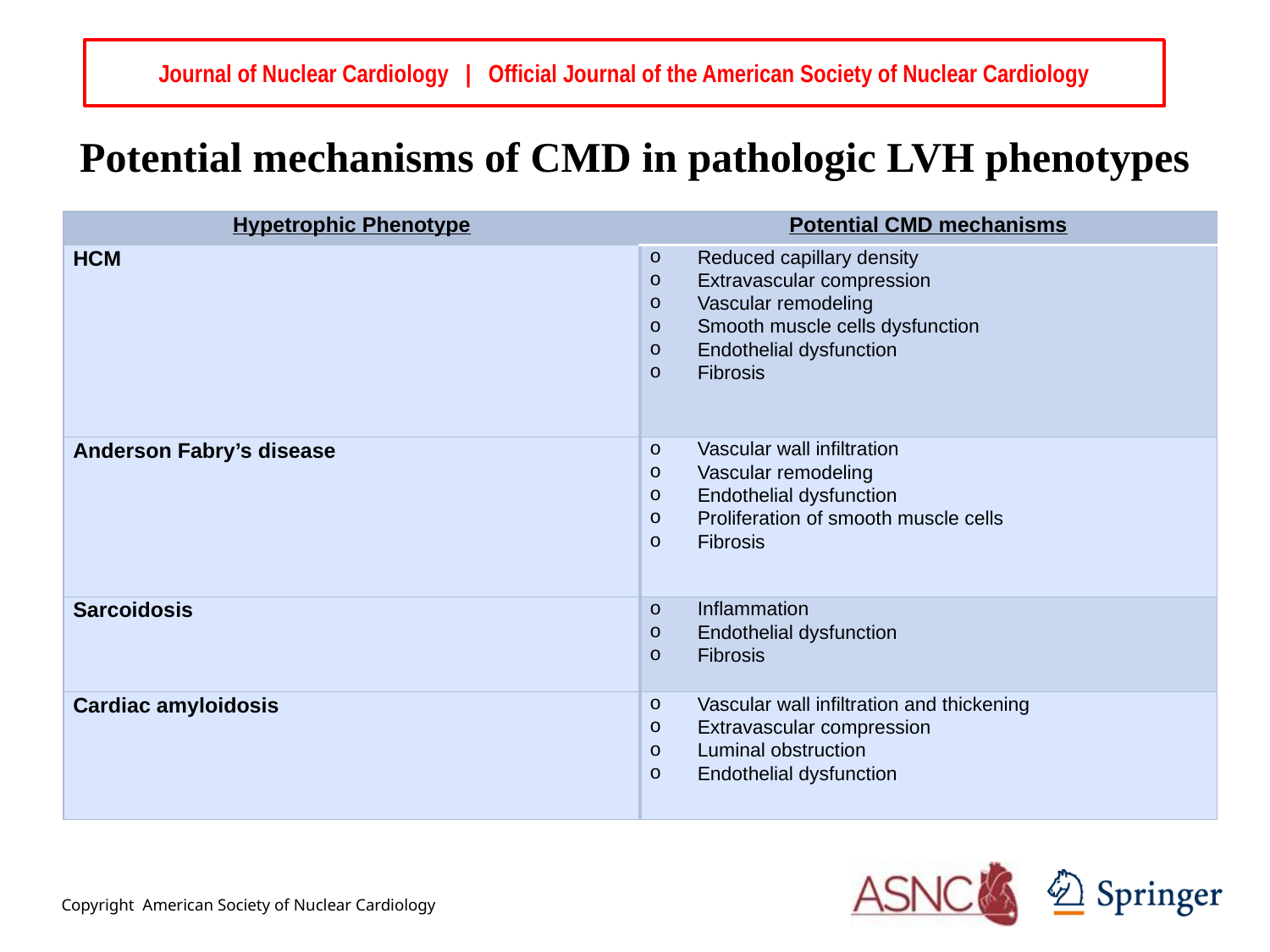

Journal of Nuclear Cardiology | Official Journal of the American Society of Nuclear Cardiology
# Potential mechanisms of CMD in pathologic LVH phenotypes
| Hypetrophic Phenotype | Potential CMD mechanisms |
| --- | --- |
| HCM | Reduced capillary density Extravascular compression Vascular remodeling Smooth muscle cells dysfunction Endothelial dysfunction Fibrosis |
| Anderson Fabry’s disease | Vascular wall infiltration Vascular remodeling Endothelial dysfunction Proliferation of smooth muscle cells Fibrosis |
| Sarcoidosis | Inflammation Endothelial dysfunction Fibrosis |
| Cardiac amyloidosis | Vascular wall infiltration and thickening Extravascular compression Luminal obstruction Endothelial dysfunction |
Copyright American Society of Nuclear Cardiology

## Slide 4
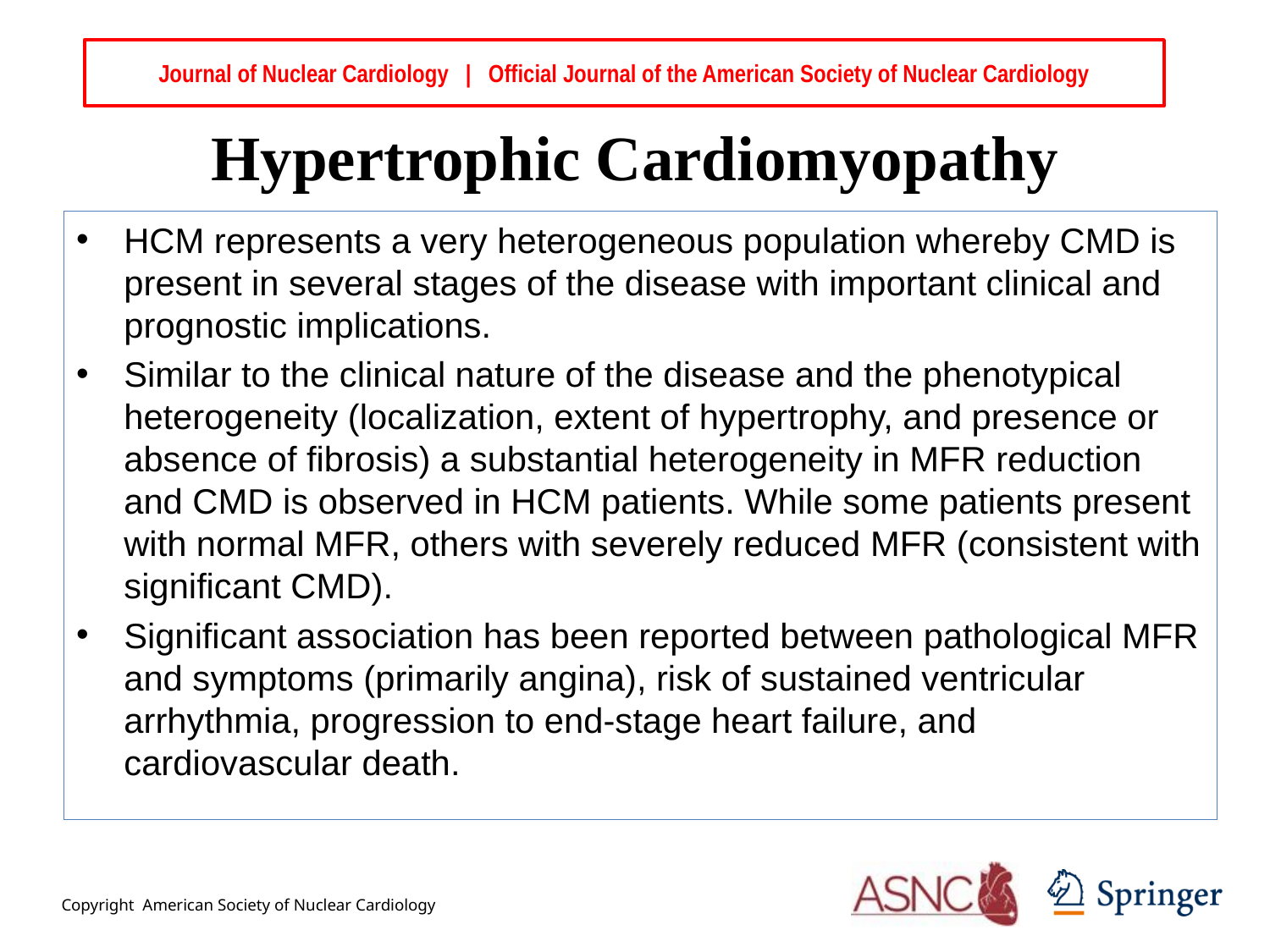

Journal of Nuclear Cardiology | Official Journal of the American Society of Nuclear Cardiology
# Hypertrophic Cardiomyopathy
HCM represents a very heterogeneous population whereby CMD is present in several stages of the disease with important clinical and prognostic implications.
Similar to the clinical nature of the disease and the phenotypical heterogeneity (localization, extent of hypertrophy, and presence or absence of fibrosis) a substantial heterogeneity in MFR reduction and CMD is observed in HCM patients. While some patients present with normal MFR, others with severely reduced MFR (consistent with significant CMD).
Significant association has been reported between pathological MFR and symptoms (primarily angina), risk of sustained ventricular arrhythmia, progression to end-stage heart failure, and cardiovascular death.
Copyright American Society of Nuclear Cardiology

## Slide 5
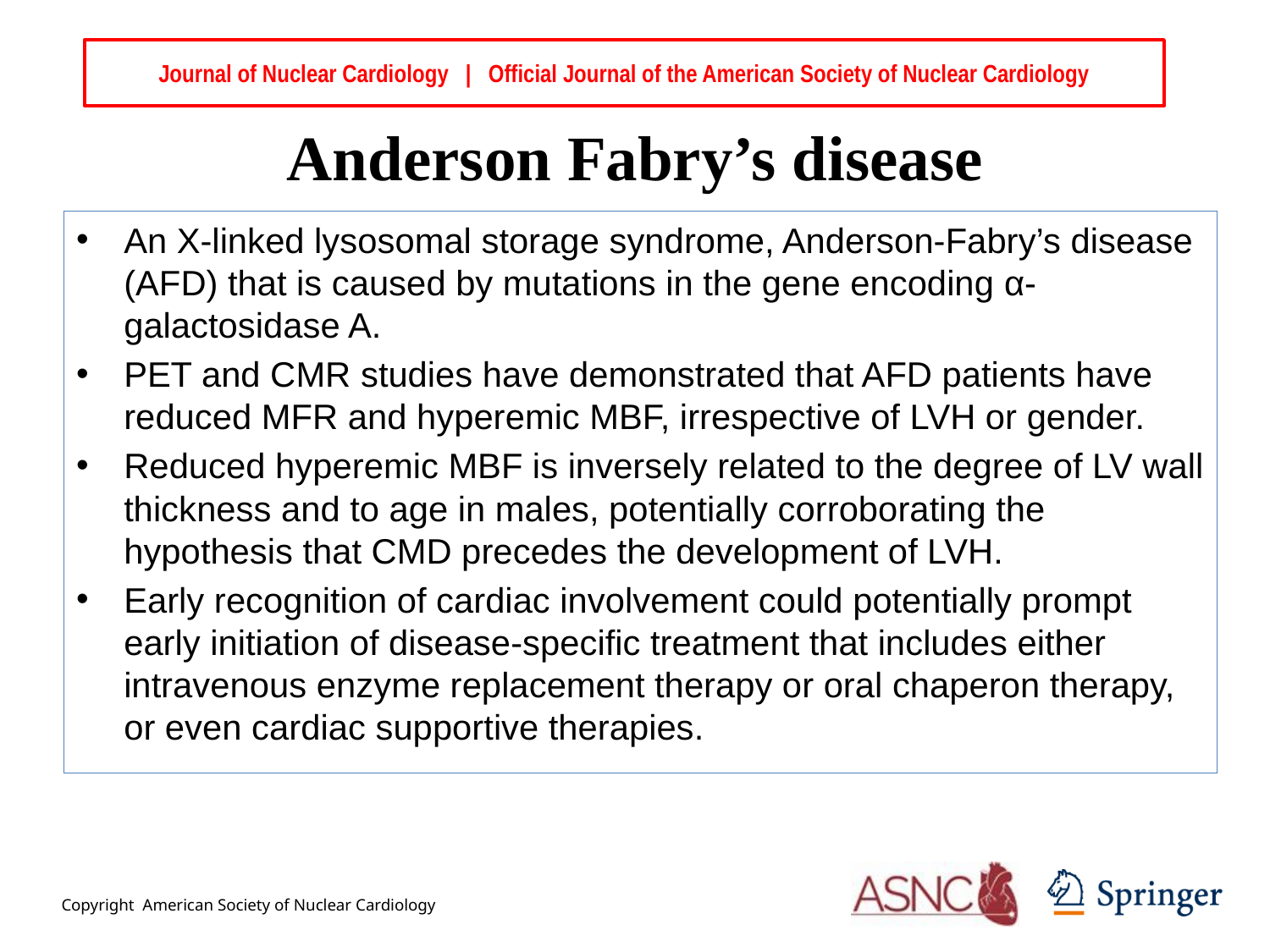

Journal of Nuclear Cardiology | Official Journal of the American Society of Nuclear Cardiology
# Anderson Fabry’s disease
An X-linked lysosomal storage syndrome, Anderson-Fabry’s disease (AFD) that is caused by mutations in the gene encoding α-galactosidase A.
PET and CMR studies have demonstrated that AFD patients have reduced MFR and hyperemic MBF, irrespective of LVH or gender.
Reduced hyperemic MBF is inversely related to the degree of LV wall thickness and to age in males, potentially corroborating the hypothesis that CMD precedes the development of LVH.
Early recognition of cardiac involvement could potentially prompt early initiation of disease-specific treatment that includes either intravenous enzyme replacement therapy or oral chaperon therapy, or even cardiac supportive therapies.
Copyright American Society of Nuclear Cardiology

## Slide 6
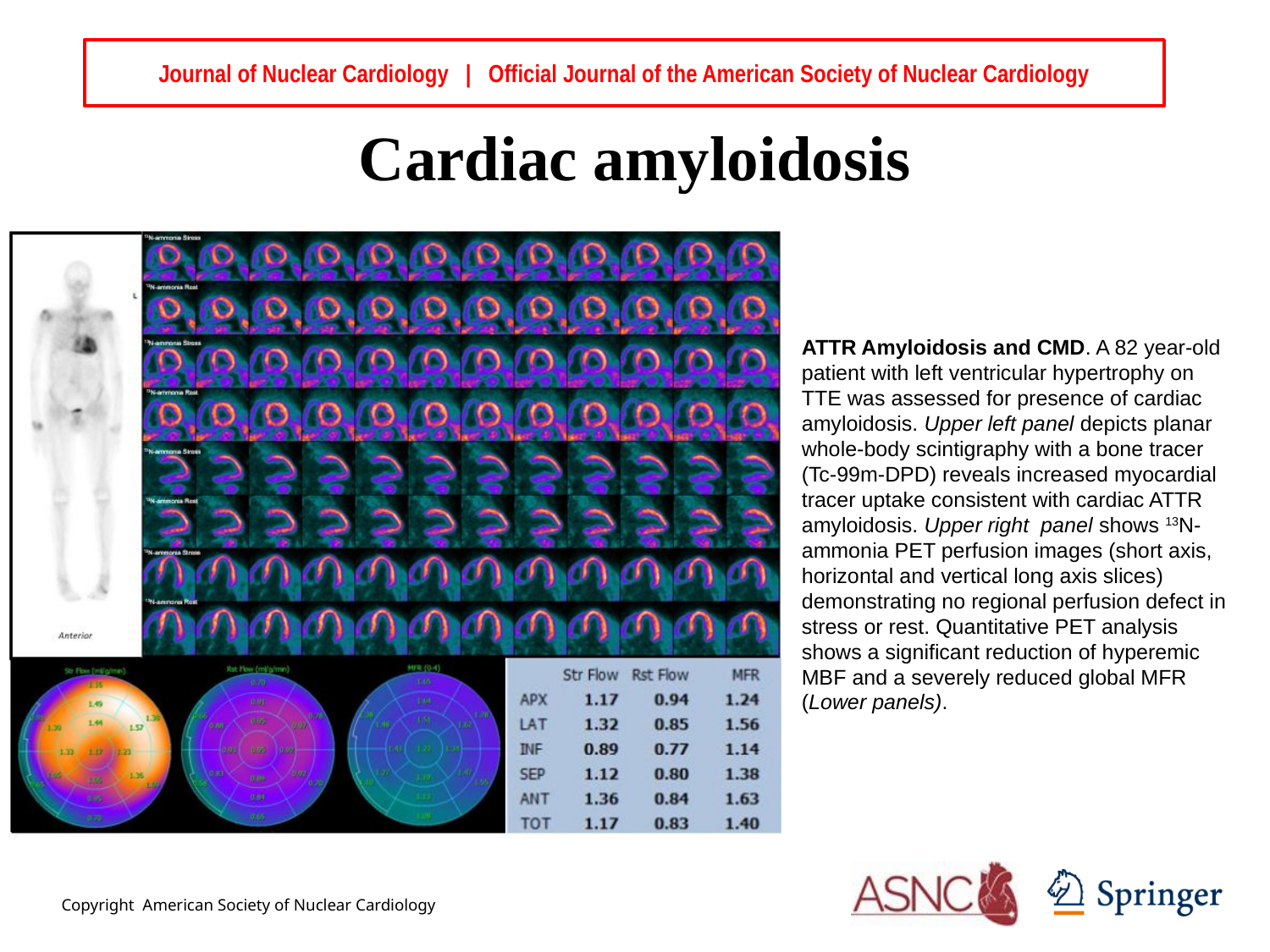

Journal of Nuclear Cardiology | Official Journal of the American Society of Nuclear Cardiology
# Cardiac amyloidosis
ATTR Amyloidosis and CMD. A 82 year-old patient with left ventricular hypertrophy on TTE was assessed for presence of cardiac amyloidosis. Upper left panel depicts planar whole-body scintigraphy with a bone tracer (Tc-99m-DPD) reveals increased myocardial tracer uptake consistent with cardiac ATTR amyloidosis. Upper right panel shows 13N-ammonia PET perfusion images (short axis, horizontal and vertical long axis slices) demonstrating no regional perfusion defect in stress or rest. Quantitative PET analysis shows a significant reduction of hyperemic MBF and a severely reduced global MFR (Lower panels).
Copyright American Society of Nuclear Cardiology

## Slide 7
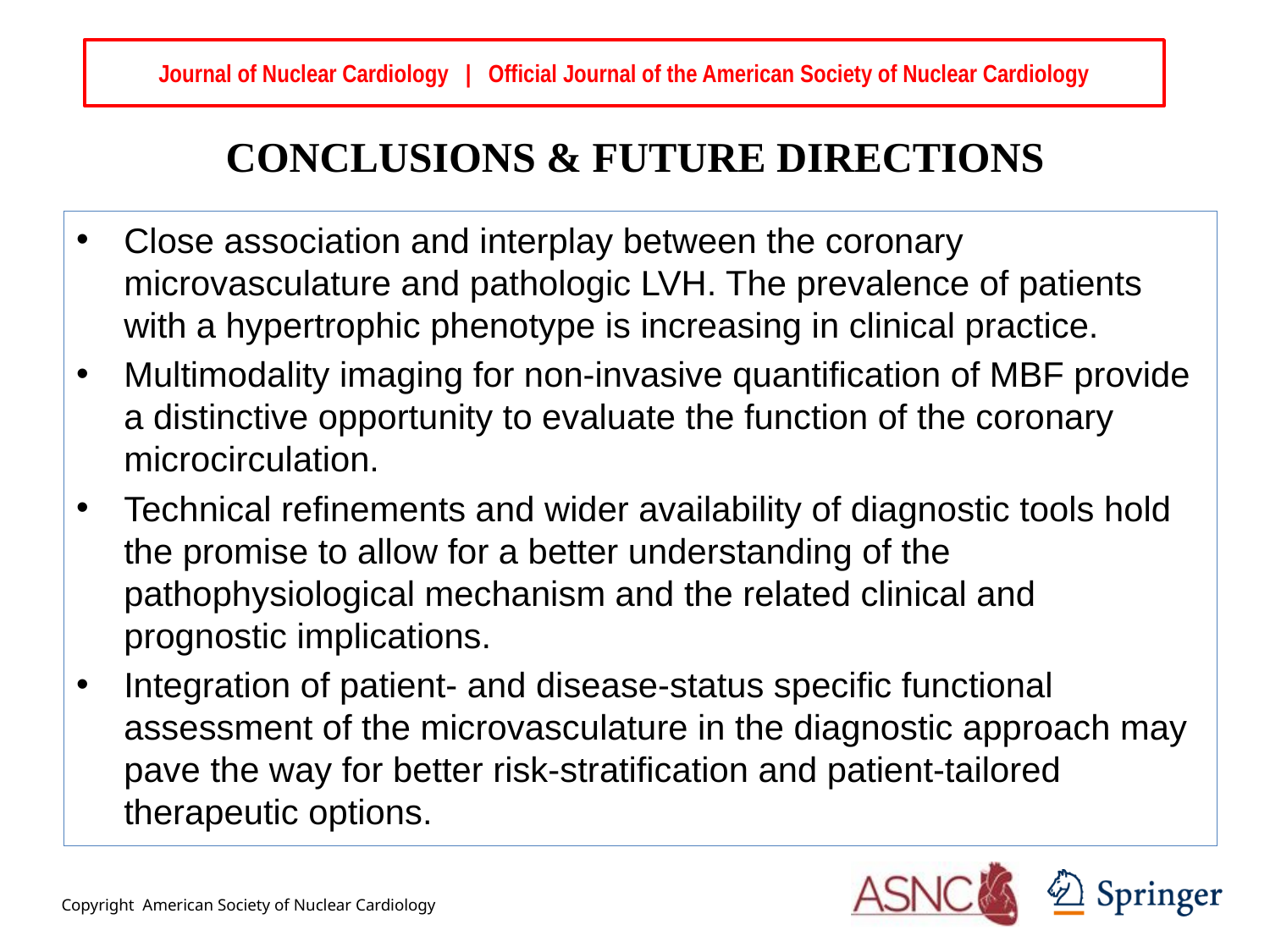

Journal of Nuclear Cardiology | Official Journal of the American Society of Nuclear Cardiology
# CONCLUSIONS & FUTURE DIRECTIONS
Close association and interplay between the coronary microvasculature and pathologic LVH. The prevalence of patients with a hypertrophic phenotype is increasing in clinical practice.
Multimodality imaging for non-invasive quantification of MBF provide a distinctive opportunity to evaluate the function of the coronary microcirculation.
Technical refinements and wider availability of diagnostic tools hold the promise to allow for a better understanding of the pathophysiological mechanism and the related clinical and prognostic implications.
Integration of patient- and disease-status specific functional assessment of the microvasculature in the diagnostic approach may pave the way for better risk-stratification and patient-tailored therapeutic options.
Copyright American Society of Nuclear Cardiology
